# When May We Advise Gonnorrheal Patients to Marry?

**Published:** 1907-01

**Authors:** W. L. Champion

**Affiliations:** Atlanta, Ga.


### ATLANTA
### Journal-Record of Medicine

Successor to Atlanta Medical and Surgical Journal, Established 1855, and Southern Medical Record, Establisihed 1870,

OWNED BY THE ATLANTA MEDICAL JOURNAL CO.

**Published Monthly,**

*Official Organ Fulton County Medical Society, State Examining Board, Presbyterian Hospital, Etc.*

VOL. VIII. JANUARY, 1907. No. 10

BENARD WOLFF, M. D., EDITOR, 319-320 Prudential Bldg.

M. B. HUTCHINS, M. D,, BUSINESS MANAGER, 1007-1008 Ceutury Bldg.

E. C. BALLENGER, M. D., ASSOCIATE EDITOR AND ASSISTANT MANAGER.

### ORIGINAL COMMUNICATIONS.

### WHEN MAY WE ADVISE GONORRHEAL PATIENTS TO MARRY.

(Read before the Medical Association of Georgia, April, 1906.)

By W. L. CHAMPION, M. D., Atlanta, Ga.

Appreciating the fact that from fifty to seventy five per cent of the acute inflammatory conditions of the female pelvis are due to gonorrheal infection, that gonorrhea in the male is as deceptive as Satan, and frequently a very troublesome disease to cure, makes the above question to the thoughtful and conscientious physician one of serious import.

When we consider this high percentage of innocent women suffering as a result of man's ignorance and depravity, and knowing that ninety or ninety-five per cent. of the male population at some period of their lives suffer from gonorrhea, this question cannot too often be brought before medical men for discussion. Patients are so frequently seen with a chronic gonorrhea which has extended over a period of years that the question is often asked, is the disease curable? When the infection is confined to the genital organs, that is, the urethra, bladder, prostate, vesicles and adjacent glandular structures ti is curable at any stage. When the kidneys or the joints and other serious membranes are attacked it is doubtful if they are ever restored to their normal condition. I warrant the assertion and can demonstrate it, that in a large majority of the chronic or relapsing cases of gonorrhea, the seat of trouble or focus of infection is in the prostate gland and seminal vesicles. Next in frequenty it will be found in the thickened and inflammatory tissues around urethral strictures or fistulous tracts and periurethral glands.

In a case of gonorrhea that has extended over a period of months the gonoccoccus has not limited its ravages to the epithelial covering of the urethra, which an ordinary injection or local application will reach, but has penetrated deeper into the tissues and therefore more stubbornly resists therapeutic measures. It is not infrequent to see a patient who has gone for months without any external evidence of the disease, and the urine containing very few "clap shreds" develop as it were a fresh attack from the passage of a full size sound; or thorough massage of the prostate will bring to the surface gonoccocci that have been lying dormant in the glands and fallicles. The point which I wish to emphasize is that the disappearance of all external evidence of the disease as a rule leads the patient and too frequently his physician to believe that he is well, when in fact he is as dangerous from the standpoint of being infectious as if he had a purulent discharge from the urethra. It is not only the genito-urinary surgeon who should be posted in regard to these matters, but every physician who takes charge of a case of gonorrhea should be well enough informed to make the neccessary examination and tests and determine whether a man can infest his wife or not.

The urine of a gonorrheal patient should be examined each day in a clean, clear glass with a good light to see if shreds or particles of pus are in the specimen, and to note the improvement or retrograde action of the disease. This test is as important as taking the pulse of a patient with an acute febrile disease. There may be pus in a patient's urine and gonoccocci absent, but it is the exception when there is a history of gonorrhea. It is the duty of a physician before dismissing a patient as cured to see that his urine is free of pus, or if pus is present, that it does not contain gonoccocci; that the prostate or seminal vesicles are not involved; that there are no fistulous tracts or enlarged glands about the fraenmun or along the urethra; he should pass a bulbous bougie or urethraemter to note if there are contractions of the urethra, with the endoscope the entire length of the urethra can be inspected to determine if any of the follicles or crypts are diseased.

To treat gonorrhea scientifically it is absolutely necessary to use the microscope. It is criminal to write prescriptions for patients, allow them to treat their own case, and with the little knowledge they have of the disease determine from external appearances when they are well.

If every practitioner will make it a rule to carefully examine the genitals of this class of patients, feel of the prostate to ascertain if it is sensitive, hard, enlarged or involved in any manner, strip the vesicles, and examine the various secretions under the miscroscope, which is a simple procedure, it will take only a short time to gain the experience which every man should have who handles cases of this kind; then he can give to his patrons the scientific advice they pay for, and to humanity that which he owes. When a patient's urine is free of pus, when the prostate and vesicles are in a normal condition, when the periurethral glands are not enlarged, when the prostatic and seminal secretion obtained by compression and urethral discharge set up by a solution of silver nitrate does not show the gonococcus present, we can state to a gonorrheal patient that he may safely marry. The highest and most unselfish duty in our professional work is the prevention of disease and the protection of the innocent. We can in part perform this duty by protecting innocent women from the ravages of this dread disease, and lessening the number of infants soon blind to the world.

